# The combined effect of long working hours and individual risk factors on cardiovascular disease: An interaction analysis

**DOI:** 10.1002/1348-9585.12204

**Published:** 2021-02-08

**Authors:** Wanhyung Lee, Jongin Lee, Hyoung‐Ryoul Kim, Yu Min Lee, Dong‐Wook Lee, Mo‐Yeol Kang

**Affiliations:** ^1^ Department of Occupational and Environmental Medicine Gil Medical Center Gachon University College of Medicine Incheon Republic of Korea; ^2^ Department of Occupational and Environmental Medicine College of Medicine Seoul St. Mary’s Hospital The Catholic University of Korea Seoul Republic of Korea; ^3^ Department of Preventive Medicine College of Medicine Seoul National University Seoul Republic of Korea

**Keywords:** cardiovascular disease, chronic disease, interaction, long working hours, unhealthy behavior

## Abstract

**Objectives:**

We examined whether the effect of long working hours on the risk of developing cardiovascular disease (CVD) differs depending on individual risk factors.

**Methods:**

Seven‐year follow‐up data were extracted from the 2009 to 2016 waves of the Korea Health Panel Survey. Physician's diagnosis or medication for hypertension, diabetes, dyslipidemia, and obesity were included as chronic disease status. Smoking, drinking alcohol, and exercise levels were considered as lifestyle behavior. Hazard ratios were calculated using the Cox regression models to evaluate the risk of CVD related to chronic diseases and unhealthy behavior, based on working hour groups, after adjusting for other covariates. The interactive effects of long working hours with or without chronic diseases and unhealthy behavior on CVD were assessed using the relative excess risk due to interaction and attributable proportion measures.

**Results:**

There was a statistically significant interaction between long working hours and chronic diseases within the population, especially among male participants (*P*‐value for interaction <.01 and .03, respectively). There were no significant interactions between unhealthy behavior and long working hours.

**Conclusions:**

Long working hours and chronic disease have a synergistic negative effect on the risk of CVD.

## 1 INTRODUCTION

The influence of working hours on health has gained global attention from the perspectives of research and public health policy. Several studies have investigated the health effects of working hours and found that long working hours are associated with a variety of health problems, including unhealthy lifestyle behavior, musculoskeletal disorders, work injuries, hypertension, diabetes, depression and suicide ideation, sleep problems, and premature birth.^1^, ^2^, ^3^, ^4^, ^5^, ^6^, ^7^, ^8^, ^9^ Kivimaki et al published the largest meta‐analysis of the relationship between working hours and cardiovascular disease (CVD).^10^ The average CVD risk ratio for those who worked more than 55 h per week compared to those working 35 to 40 h per week was 1.13 [95% CI: 1.02‐1.26], and the risk ratio of incident stroke was 1.33 (95% CI: 1.11‐1.61).

Plenty of evidence from well‐designed observational studies and clinical trials have shown that chronic diseases such as diabetes, hypertension, dyslipidemia, and obesity, and unhealthy behavior including smoking, drinking, and physical inactivity, can influence the risk of CVD.^11^, ^12^, ^13^ These individual risk factors are frequently and consistently reported among workers with long working hours.^3^, ^14^ Although several researchers have speculated that the mediating effect of these risk factors may explain the relationship between long working hours and increased CVD risk,^1^, ^8^, ^15^ the evidence is scarce. Therefore, when determining work‐relatedness for workers' compensation claims, it is rarely approved as an occupational disease if there are individual risk factors, even if the claimant's working hours are long enough.

Until now, no previous study has examined the combined effect of long working hours and individual risk factors or evaluated their potential interactions with CVD. Therefore, in this study, we examined whether the effect of long working hours on the risk of developing CVD differs depending on the existence of individual risk factors.

## 2 METHODS

### 2.1 Study design and data collection

**FIGURE 1:**
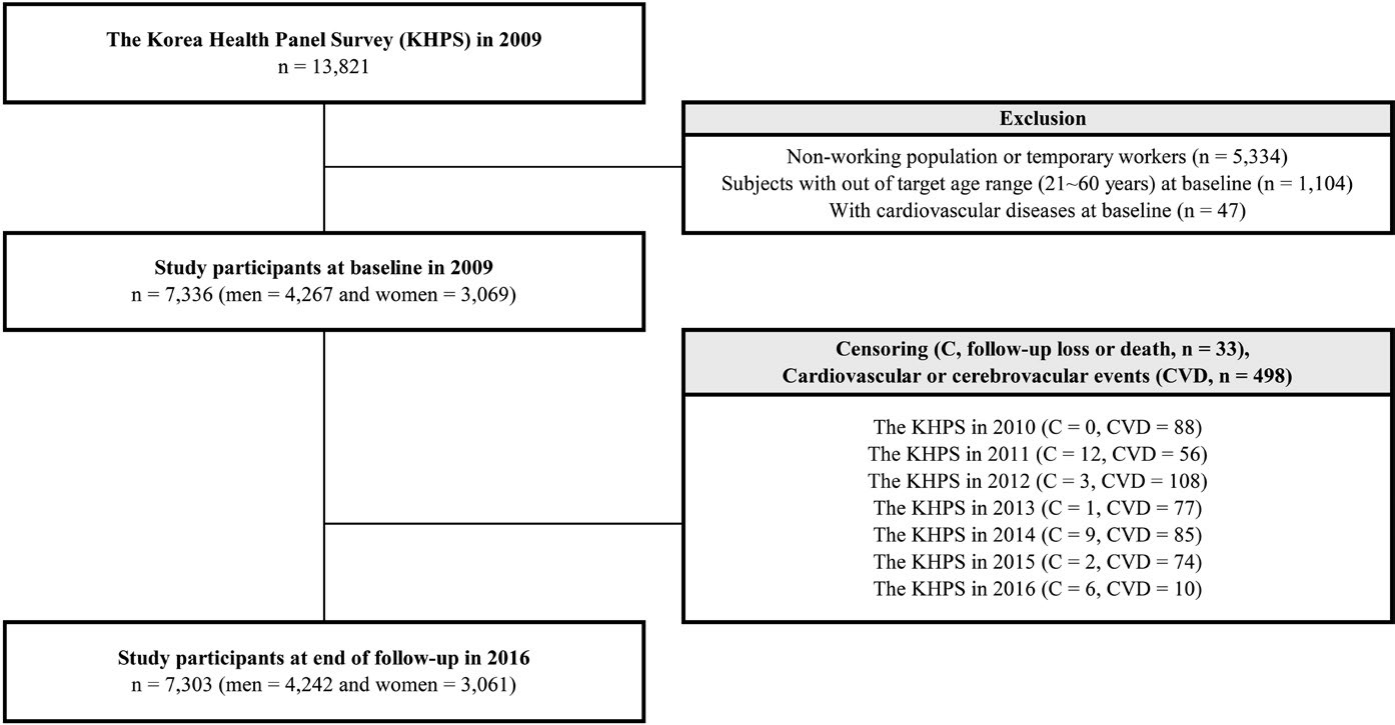
Schematic diagram depicting study population

The Korea Health Panel Survey (KHPS) provides detailed information on health insurance and identifies the longitudinal state of healthcare service use for a nationally representative Korean population. The KHPS is conducted annually by the Korea Institute for Health and Social Affairs and the National Health Insurance Service since 2008. For the purpose of national representation, the KHPS sampled subjects based on a two‐step probability proportional stratified colony extraction method using approximately 8000 households from 350 survey areas from among 90% of the 2005 population and housing census data. The KHPS is conducted through questionnaires by trained interviewers by gathering data from participants on their socioeconomic status, medical expenditure, the status of chronic diseases, emergency facility use history, in‐ or out‐patient history, and private health insurance. All the participants enrolled in the KHPS agreed to participate in further scientific research. All data and detailed cohort profiles are freely accessible on the website (https://www.khp.re.kr:444/eng/main.do) for researchers or investigators. For this study, 7‐year follow‐up data were collected from the 2009 to 2016 waves of the KHPS, excluding 2008 due to limited information about working conditions. The study participants and exclusion criteria are shown in Figure 1. From the 2009 KHPS, participants (n = 13 821) who were not working or were temporary workers (n = 5334), who were out of the target age range (21‐60 years) at baseline (n = 1104), and those with CVD at baseline (n = 47) were excluded. Finally, a total of 7336 workers were included in the current analysis. During the 7‐year follow‐up period, 33 subjects who were lost to follow‐up or had died (except due to CVD‐related disease) were censored. Finally, 7303 participants remained at the end of the follow‐up period in 2016.

### 2.2 Ethical approval

The authors assert that all procedures contributing to this work comply with the ethical standards of the relevant national and institutional committees on human experimentation and with the Helsinki Declaration of 1975, as revised in 2008. All participants signed a consent form, and anonymity and confidentiality were assured. Ethics approval for this study was not required because this was a retrospective analysis of national surveillance data that is free of personally identifiable information.

### 2.3 CVD and long working hours

The CVD status was assessed using a death certificate and a questionnaire answered by self or family members. Death from cardiovascular or cerebrovascular diseases was defined as cause‐specific deaths occurring during the follow‐up period using the International Classification of Diseases, 10th Revision (ICD‐10), and Diseases of the circulatory system (I00‐I99). Newly onset cardiovascular or cerebrovascular diseases during the follow‐up period were defined using hospital visit history with ICD‐10, ischemic heart diseases (I20‐I25), and cerebrovascular diseases (I60‐I69).

To define long working hours, we used the following self‐reported question: “How many hours do you work at your job per week on average, including overtime but excluding mealtimes?” Long working hours were defined by the Standard Labour Act in the Republic of Korea. The Standard Labour Act includes regulation of weekly working hours, which allows up to 40 working hours per week, with an additional 12 h per week. However, it can be extended with the agreement of all parties. Thus, long working hours included working over 52 h per week at baseline.

### 2.4 Chronic diseases and health behavior

The KHPS included information about the participants’ medical history. Physician's diagnosis or medication for hypertension, diabetes, dyslipidemia, and obesity (body mass index ≥25 kg/m^2^) were included as chronic disease status. We used information about health behavior from the KHPS in 2009.

Smoking, drinking alcohol, and exercise levels were considered as lifestyle behavior. Unhealthy behavior was indicated by a current smoking or drinking problem and a lack of exercise. Current smoking included those who had the habit of smoking during the time of the survey and those who smoked more than 100 cigarettes in their lifetime. An alcohol drinking problem was defined as the consumption of >40 and >60 g of alcohol per day, more than twice per week, for female and male participants, respectively. The subjects who exercised fewer than once per week were classified as the non‐exercise group.

According to the number, chronic diseases or unhealthy behavioral factors were classified into two groups: “0” and “≥1.”

### 2.5 Other covariates

In this study, age, sex, educational status, and household income level were used as socioeconomic variables. Educational level was categorized into three groups: elementary to middle school, high school, and graduate/college or higher. Household income level was categorized using quintiles (one being the lowest and five being the highest) based on annual income. The type of work indicated employment status and was divided into two categories: paid workers and self‐employed. Occupations were categorized into four groups based on the 10 major categories set by the International Standard Classifications of Occupations: white‐collar (managers, professionals, technicians, and associate professionals), pink‐collar (clerical support, service, and sales workers), green‐collar (skilled agricultural, forestry, and fishery workers), and blue‐collar (craft and related trades, plant and machine operators and assemblers, and elementary occupations).^16^

### 2.6 Statistical analysis

The incidence of CVD was calculated for each variable, and a chi‐square test was used to evaluate the relationship between each variable and CVD. The survival time was defined as the time between the date of the first survey wave and the day CVD occurred. Hazard ratios (HR) and 95% CI were calculated using Cox regression models to evaluate the risk of CVD associated with chronic diseases and unhealthy behavior based on working hour groups, after adjusting for age, educational level, household income level, type of work, and occupational classification. The interactive effects of long working hours, with or without chronic diseases (undergoing diagnosis or treatment of hypertension, diabetes, and dyslipidemia, and obesity), or unhealthy behavior (current smoking, problem drinking, and no exercise) on CVD were estimated. To describe the adjunctive interaction effect, we used the relative excess risk due to interaction (RERI) and attributable proportion (AP) measures. The RERI and AP were calculated from the HR of CVD with strata of exposure to long working hours and chronic diseases or unhealthy behavioral status, using those without long working hours and chronic diseases or unhealthy behavior as reference group. Values over zero of RERI and AP indicated a positive interaction effect. To reduce the potential effect of working hour change over the follow‐up periods, we further conducted subgroup analysis by survey years of working hours and calculated pooled estimates from them. All statistical analyses were performed using SAS (version 9.6; SAS Institute, Cary, NC, USA). Two‐tailed *P*‐values < .05 were considered indicative of statistical significance.

## 3 RESULTS

Figure 1 shows the criteria for study participants and the follow‐up profile. At the baseline in 2009, a total of 7336 working individuals were selected for the current analysis. During the 7‐year follow‐up period, 33 patients were lost to follow‐up or had died (from non‐CVD‐related diseases). Finally, 7303 participants remained at the end of the follow‐up period in 2016 (the follow‐up rate was 99.5% from 2009 to 2016).

The baseline characteristics based on CVD are presented in Table 1. The incidence of CVD was 6.8% during the follow‐up period, and 7.6% and 5.6% among male and female workers, respectively. The older population had an increased incidence of CVD. Lower educational and income levels were also related to a higher incidence of CVD, although only the educational level had greater significance. Self‐employed and green‐collar workers showed a significantly increased incidence of CVD. The long working hour group had a higher incidence of CVD, without statistical significance. Subjects with hypertension, diabetes, and obesity had an increased incidence of CVD, with statistical significance. Unhealthy behavior did not appear to impact the incidence of CVD.

**TABLE 1:** Baseline characteristics of the study participants at baseline according to cardiovascular and cerebrovascular diseases or death (CVD) during follow‐up periods

	CVD		*P* value
	No	Yes	
All participants	6838	498	
Socioeconomic status			
Age (y)			<.0001
21‐30	659 (9.6)	5 (1.0)	
31‐40	1648 (24.1)	39 (7.8)	
41‐50	2187 (32.0)	146 (29.3)	
51‐60	2344 (34.3)	308 (61.8)	
Sex			.0009
Men	3942 (57.6)	325 (65.3)	
Women	2896 (42.4)	173 (34.7)	
Educational status			<.0001
Middle school	1528 (22.3)	201 (40.4)	
High School	629 (38.4)	176 (35.3)	
College or higher	2681 (39.2)	121 (24.3)	
Yearly household income (Quintile)			.7437
1st	296 (4.3)	26 (5.2)	
2nd	1062 (15.5)	60 (12.0)	
3rd	1570 (23.0)	127 (25.5)	
4th	1897 (27.7)	156 (31.3)	
5th	2013 (29.4)	129 (25.9)	
Type of work			.0321
Paid workers	4907 (71.8)	335 (62.0)	
Self‐employed	1931 (28.2)	163 (32.7)	
Occupational classification			<.0001
White‐collar	2406 (30.6)	121 (36.3)	
Pink‐collar	1501 (14.0)	112 (17.3)	
Green‐collar	368 (6.3)	47 (8.0)	
Blue‐collar	2563 (27.5)	218 (40.0)	
Long working hours (weekly working >52 h)			.2277
No	4426 (64.7)	309 (62.0)	
Yes	2412 (35.3)	189 (38.0)	
Chronic diseases			
Hypertension	2090 (30.6)	181 (36.3)	.0071
Diabetes	957 (14.0)	86 (17.3)	0434
Dyslipidemia	428 (6.3)	40 (8.0)	.1181
Obesity	1881 (27.5)	199 (40.0)	<.0001
Unhealthy behaviors			
Current smoking	2111 (30.9)	162 (32.5)	.4397
With problem drinking	1541 (22.5)	119 (23.9)	.4839
No exercise	4177 (61.1)	318 (63.9)	.2205

Table 2 demonstrates the results of the Cox regression for risk of CVD related to chronic diseases and unhealthy behavior and long working hour groups. A significantly increased risk of CVD related to obesity was found in participants who had long working hours as well as in those who did not (HR [95% CI]: 1.85 [1.39‐2.47] and 1.35 [1.06‐1.70], respectively) even after adjusting for age, educational level, household income level, type of work, and occupational classification. However, only the long working hours group showed a statistically increased risk for CVD related to chronic diseases (1.45 [1.17‐1.79]). Unhealthy behavior, including current habit of smoking, was closely associated with CVD only in the group who did not have long working hours in the adjusted model. After gender stratification, there was a significantly increased risk for CVD related to obesity among male workers only (1.96 [1.37‐2.81]). Lack of exercise was significantly related to CVD among female workers who did not have long working hours. The results from pooled analyses of subgroup Cox regression by survey years showed a more prominent combined influence of long working hours and chronic disease on the risk of CVD (Table S1).

**TABLE 2:** Results of Cox regression for risk of cardiovascular and cerebrovascular diseases or death (CVD) related with baseline chronic diseases and unhealthy behaviors

	Number of participants or cases		Hazard ratio (95% confidence interval) of CVD			
	Long working hours		Crude model		Adjusted model	
			Long working hours		Long working hours	
	No	Yes	No	Yes	No	Yes
All participants	4735	2601				
Chronic diseases						
Hypertension	1484	787	**1.31 (1.04‐1.65)**	1.25 (0.93‐1.69)	1.15 (0.91‐1.45)	1.08 (0.80‐1.46)
Diabetes	679	364	1.16 (0.86‐1.57)	**1.47 (1.02‐2.11)**	0.87 (0.64‐1.19)	1.07 (0.74‐1.54)
Dyslipidemia	294	174	1.23 (0.80‐1.88)	1.40 (0.85‐2.31)	0.81 (0.53‐1.25)	0.94 (0.57‐1.55)
Obesity	1275	805	**1.51 (1.20‐1.91)**	**2.02 (1.52‐2.69)**	**1.35 (1.06‐1.70)**	**1.85 (1.39‐2.47)**
Per chronic diseases			**1.44 (1.23‐1.69)**	**1.80 (1.47‐2.20)**	1.14 (0.97‐1.34)	**1.45 (1.17‐1.79)**
Unhealthy behaviors						
Current smoking	1287	986	**1.33 (1.05‐1.69)**	0.78 (0.56‐1.04)	**1.28 (1.01‐1.62)**	0.91 (0.68‐1.27)
Problem drinking	970	690	1.08 (0.83‐1.42)	1.05 (0.77‐1.45)	1.04 (0.80‐1.37)	1.04 (0.76‐1.44)
No exercise	2935	1560	1.09 (0.86‐1.37)	1.19 (0.88‐1.60)	1.15 (0.91‐1.45)	1.17 (0.87‐1.57)
Per unhealthy behavior			**1.15 (1.00‐1.31)**	0.99 (0.84‐1.17)	**1.17 (1.02‐1.34)**	1.03 (0.87‐1.22)
Male workers	2497	1770				
Chronic diseases						
Hypertension	766	517	**1.43 (1.08‐1.89)**	1.24 (0.85‐1.80)	1.25 (0.94‐1.66)	1.06 (0.73‐1.45)
Diabetes	330	222	1.06 (0.71‐1.57)	**1.66 (1.05‐2.61)**	0.81 (0.54‐1.21)	1.21 (0.76‐1.92)
Dyslipidemia	129	100	1.07 (0.85‐1.96)	**1.89 (1.04‐3.43)**	0.77 (0.41‐1.40)	1.26 (0.69‐2.30)
Obesity	856	593	1.24 (0.94‐1.65)	**1.88 (1.32‐2.69)**	1.16 (0.87‐1.53)	**1.96 (1.37‐2.81)**
Per chronic diseases			**1.31 (1.08‐1.58)**	**1.85 (1.44‐2.38)**	1.14 (0.94‐1.39)	**1.60 (1.23‐2.07)**
Unhealthy behaviors						
Current smoking	1212	942	1.03 (0.78‐1.35)	0.77 (0.54‐1.10)	1.17 (0.89‐1.54)	0.93 (0.65‐1.33)
Problem drinking	871	627	0.92 (0.69‐1.23)	1.31 (0.92‐1.88)	0.94 (0.70‐1.26)	1.26 (0.88‐1.81)
No exercise	1273	956	1.06 (0.80‐1.39)	1.07 (0.75‐1.53)	1.09 (0.83‐1.44)	1.05 (0.74‐1.51)
Per unhealthy behavior			1.00 (0.87‐1.16)	1.02 (0.84‐1.24)	1.08 (0.93‐1.25)	1.06 (0.87‐1.30)
Female workers	2238	831				
Chronic diseases						
Hypertension	718	270	1.14 (0.77‐1.70)	1.26 (0.77‐2.07)	1.03 (0.69‐1.53)	1.15 (0.70‐1.89)
Diabetes	349	142	1.43 (0.90‐2.29)	1.17 (0.64‐2.15)	1.06 (0.66‐1.69)	0.89 (0.48‐1.63)
Dyslipidemia	165	74	1.63 (0.89‐2.96)	0.82 (0.33‐2.03)	0.96 (0.52‐1.76)	0.58 (0.23‐1.46)
Obesity	419	212	**1.74 (1.14‐2.66)**	**2.44 (1.51‐3.95)**	1.15 (0.75‐1.78)	1.38 (0.83‐2.27)
Per chronic diseases			**1.63 (1.23‐2.15)**	**1.70 (1.21‐2.39)**	1.10 (0.82‐1.47)	1.12 (0.77‐1.61)
Unhealthy behaviors						
Current smoking	75	44	0.56 (0.14‐2.25)	0.85 (0.27‐2.68)	0.32 (0.08‐1.32)	0.88 (0.28‐2.80)
Problem drinking	99	63	NA	0.18 (0.03‐1.29)	NA	0.18 (0.03‐1.27)
No exercise	1662	604	**2.14 (1.24‐3.70)**	1.45 (0.80‐2.61)	**2.12 (1.22‐3.67)**	1.66 (0.92‐2.99)
Per unhealthy behavior			1.23 (0.86‐174)	0.99 (0.66‐1.49)	1.16 (0.82‐1.65)	1.05 (0.72‐1.52)

Note

Bold indicates statistical significance. Adjusted model: Adjusted for age, educational level, household income level, type of work, and occupational classification. NA: not available due to the insufficient number of cases in the strata to construct the Cox‐proportional hazard model. All results were from referred counter‐group of each category (no hypertension, no diabetes, no dyslipidemia, no obesity, past/never smoking, social/no drinking, or regular exercise).

**FIGURE 2:**
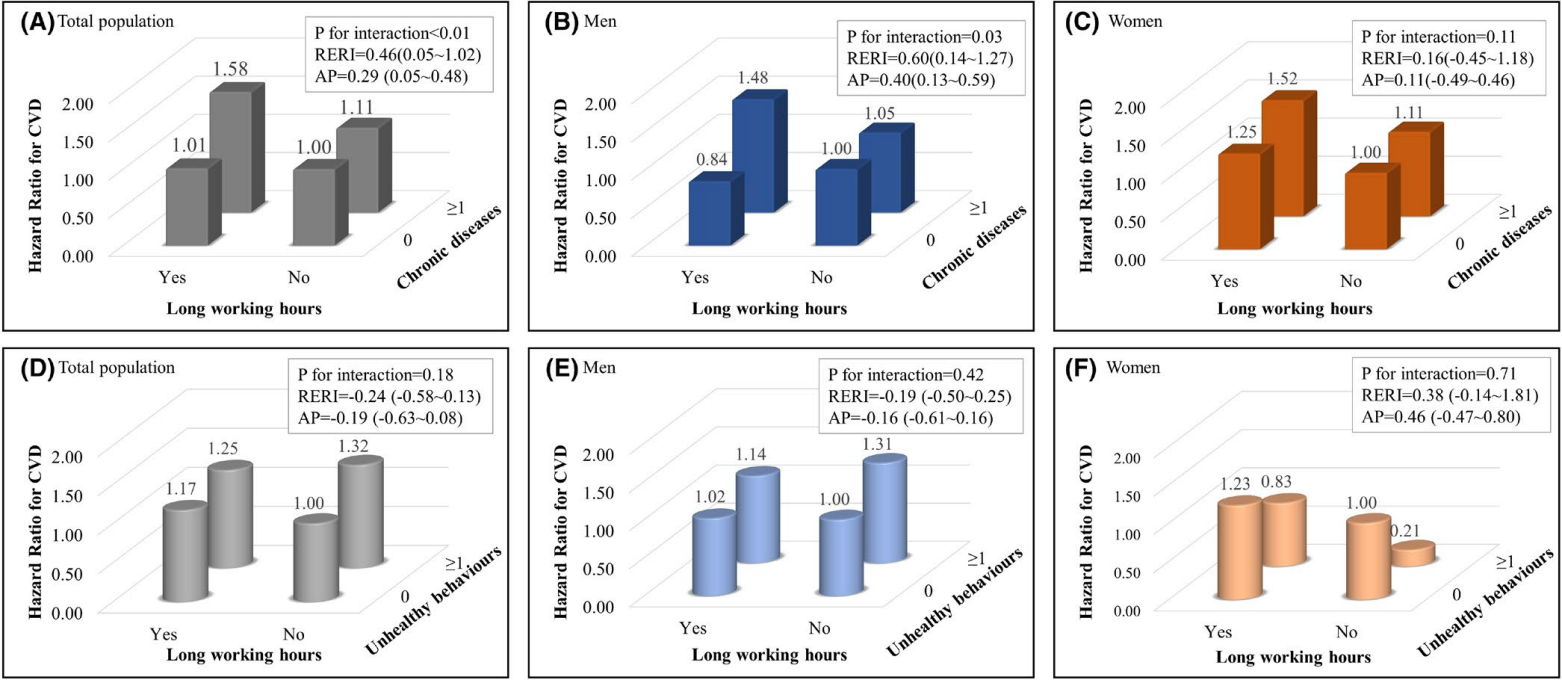
Results of interaction analysis of long working hours with chronic diseases or unhealthy behaviors on cardiovascular and CVD. Multivariable adjusted for age, educational level, household income level, type of work, and occupational classification. AP, attributable proportion (95% CI); CVD, cardiovascular and cerebrovascular diseases or death; RERI, relative excess risk (95% CI) due to interaction. Chronic diseases indicate total count of chronic diseases including the diagnosis or treatment of hypertension, diabetes, dyslipidemia, and obesity (body mass index ≥ 25), divided into two groups: no chronic disease and one or more chronic diseases. Unhealthy behaviors indicate the total count of unhealthy behaviors such as current smoking, problem drinking, and no exercise, divided into two groups: no unhealthy behaviors and one or more unhealthy behaviors

Figure 2 shows the results of the interaction analysis of long working hours with chronic diseases or unhealthy behavior on CVD with gender stratification. There was a statistically significant interaction between long working hours and chronic diseases in the population, especially male participants (*P*‐value for interaction < .01 and .03, respectively). The estimations of RERI were 0.46 (95% CI = 0.05‐1.02) for the total population and 0.60 (95% CI = 0.14‐1.27) for male workers. The estimations of AP were 0.29 (95% CI = 0.05‐0.48) for the total population and 0.40 (95% CI = 0.13‐0.59) for males. There were no significant interactions between unhealthy behavior and long working hours.

## 4 DISCUSSION

This study investigated the interaction between long working hours and the individual risk factors of chronic disease and unhealthy behavior on CVD. In a longitudinal survey of a nationally representative Korean population, the combination of long working hours and chronic diseases such as diabetes, hypertension, dyslipidemia, and obesity jointly influenced the risk of CVD. The AP was 40%. The combined effect of unhealthy behavior, such as smoking, problem drinking, and physical inactivity, was not statistically significant.

The results of our study suggest a synergistic interaction between long working hours and chronic disease on the risk of CVD. If obese men worked longer than 52 h per week, they had 1.88 times the increased risk of CVD compared to obese men who did not work long hours. No previous studies reported on the possible interaction effects between CVD and working hours associated with chronic diseases; however, a study examined the moderating effects on the relationship between working hours and subjective well‐being. Pereira and Coelho investigated several moderators of the relationship between work hours and well‐being.^17^ The results showed that the negative relationship between working hours and subjective well‐being is worse for older and less healthy individuals. Hence, the authors suggest that there is a need to redesign the work schedules of older and less healthy employees. Virtanen tested the interaction of lifestyle behavioral factors (including sleeping hours, smoking, alcohol use, daily fruit and vegetable consumption, and exercise) with working hours, predicting coronary heart disease using data from the longitudinal Whitehall II study. The findings, however, suggested no interaction effects.^18^ These results are concurrent with the findings of our study.

Meanwhile, it has been argued that working long hours not only directly affects the health of workers, but can also lead to maladaptive behavior such as smoking, drinking, and lack of physical activity.^1^, ^3^, ^14^ In turn, unhealthy behavior causes physiological changes (eg, high blood pressure, high blood glucose and cholesterol levels, and weight gain) and chronic diseases (eg, hypertension, diabetes, dyslipidemia, and obesity), eventually resulting in CVD. Therefore, it should be noted that these processes are not mutually exclusive and may operate simultaneously.

Our research also explored gender differences in the interactive effect between individual risk factors and long working hours on the risk of CVD. We found significant results among male participants, but this was not the case among female participants. The reason for these differences with respect to gender may be due to the existing influence of the male breadwinner—female homemaker model. The traditional gender‐role stereotype assumes that the husband is primarily responsible for the family income, and thus he has greater value at work, while the wife is more responsible for family matters. In particular, East Asian culture encourages husbands to spend more time on the job to maximize the family's economic benefits.^19^ According to this ethic of having separate gender roles, husbands work overtime and the temporary sacrifice of family life is perceived as “acceptable,” even when the risk of disease is high. However, women tend to have low acceptance thresholds for long working hours, and if they are unhealthy they are more likely to give up their desire to work before the deterioration of their health.^4^ Another possible reason for the lack of statistical significance among female participants might be methodological, due to a smaller sample size or biased distribution of jobs with relatively weak labor intensity.

To the best of our knowledge, this is the first study to investigate how long working hours and other individual risk factors have a combined effect on the risk of CVD. One of the major strengths of our study is the prospective design with long‐term follow‐up. However, there are also some limitations. First, as information on working hours, chronic diseases, and unhealthy behavior were based on self‐reports, there is a potential for information bias. Second, our estimates of combined effect and synergistic interactions were assessed on a relative scale, derived from the widely used Cox proportional hazards model. As the Cox model relied on the proportional hazards assumption, if time‐varying confounders were present, it could be biased. Particularly, working situation (including working hours) generally changes often, so the baseline information could not explain the changeable situations during the 7‐year follow‐up period. Therefore, the results of our analysis should be interpreted carefully, considering the potential effect of this misclassification on our findings and the possibility that the misclassification may violate the conclusion. When we conducted subgroup analysis by survey years of working hours and obtained the pooled risk estimates from them, there was no difference in the conclusion. (Table S1). Third, chronic diseases and unhealthy behavior were simply categorized as “0” or “≥1,” because if the categories are subdivided, the frequency of each category becomes very small, which leads to instability in the statistical analysis. As a result, the opportunity to study the interaction between CVDs and each factor was sacrificed. Fourth, the selection of participants focused on the working population from the sampled survey, which could lead to selection bias as the KHPS was not established to represent the working population per se, but the general Korean population. Considering that there were few data sources representative of the working population in Korea, the KHPS was deemed fit for the current analysis. Lastly, the participants were restricted to South Korea, which limits the generalizability of our findings to other populations, particularly other racial or ethnic groups. To enhance the external validity of the study, an additional analysis using a similar survey among different populations should be conducted in the future.

## 5 CONCLUSION

In conclusion, long working hours and chronic diseases have a synergistic effect on the risk of CVD. From a public health standpoint, it is important to prevent long working hours, particularly for those who have chronic diseases. Another important implication of this study is that long working hours and unhealthy behavior have a non‐significant interaction; therefore, the work‐relatedness of some workers with CVD is undeniable, even if they have unhealthy behavior. Further studies are warranted to confirm our findings and clarify the underlying mechanisms using a different approach such as the repeat measurement method.

## DISCLOSURES

*Approval of the research protocol:* Ethics approval for this study was not required because this was a retrospective analysis of national surveillance data that is free of personally identifiable information. *Informed consent:* All participants signed a consent form, and anonymity and confidentiality were assured. *Registry and the registration no. of the study/trial:* N/A. *Animal studies:* N/A. *Conflict of interest:* The authors declare no conflicts of interest.

## AUTHOR CONTRIBUTIONS

MYK and WL conceived of and designed the study. WL conducted statistical analyses. MYK and WL drafted the manuscript. MYK, JL, HRK, YML, DWL, and MYK interpreted the data and provided critical revision of the manuscript. MYK supervised the study.

## Supporting information

Table S1
