# Supplementary material for: The combined effect of long working hours and individual risk factors on cardiovascular disease: An interaction analysis
Source: J Occup Health. 2021 Feb 8;63(1):e12204. doi: 10.1002/1348-9585.12204 (PMC7870363; doi:10.1002/1348-9585.12204)
Supplement: Supplementary file 1 — Table S1 [file JOH2-63-e12204-s001.docx]

| **Supplementary Table 1.** Results from pooled analyses of subgroup Cox regression by survey years | | |
| --- | --- | --- |
|  | Adjusted Hazard Ratio (95% Confidence interval) of CVD | |
|  | Long working hours during follow-up periods | |
|  | No | Yes |
| **Person-year** | 18,530 | 11,902 |
| **Chronic diseases** |  |  |
| Hypertension | 1.05 (0.93-1.18) | **1.35 (1.15-1.58)** |
| Diabetes | 1.48 (0.66-1.92) | **1.23 (1.01-1.50)** |
| Dyslipiaemia | 0.97 (0.58-1.23) | 0.83 (0.61-1.13) |
| Obesity | **1.35 (1.20-1.52)** | **2.40 (2.06-2.81)** |
| Per chronic diseases | 1.05 (0.97-1.15) | **1.94 (1.72-2.18)** |
| **Unhealthy behaviors** |  |  |
| Current smoking | **1.41 (1.24-1.59)** | **1.35 (1.15-1.58)** |
| Problem drinking | 0.96 (0.84-1.11) | **1.54 (1.32-1.81)** |
| No exercise | 1.08 (0.96-1.22) | **1.34 (1.15-1.58)** |
| Per unhealthy behavior | **1.13 (1.06-1.21)** | **1.34 (1.23-1.46)** |
| **Male workers** |  |  |
| **Chronic diseases** |  |  |
| Hypertension | 1.09 (0.94-1.25) | **1.35 (1.13-1.63)** |
| Diabetes | 0.83 (0.67-1.01) | **1.26 (1.01-1.59)** |
| Dyslipidemia | 0.82 (0.62-1.08) | 1.05 (0.74-1.48) |
| Obesity | **1.32 (1.15-1.52)** | **2.78 (2.32-3.34)** |
| Per chronic diseases | **1.10 (1.01-1.21)** | **2.20 (1.92-2.52)** |
| **Unhealthy behaviors** |  |  |
| Current smoking | 1.24 (1.08-1.42) | **1.45 (1.20-1.74)** |
| Problem drinking | 0.88 (0.71-1.05) | **1.74 (1.45-2.09)** |
| No exercise | 1.11 (0.97-1.28) | **1.29 (1.08-1.55)** |
| Per unhealthy behavior | 1.04 (0.97-1.13) | **1.41 (1.30-1.56)** |
| **Female workers** |  |  |
| **Chronic diseases** |  |  |
| Hypertension | 1.02 (0.81-1.28) | 1.26 (0.92-1.73) |
| Diabetes | 0.78 (0.58-1.05) | 1.12 (0.76-1.65) |
| Dyslipidemia | 0.70 (0.47-1.06) | 0.55 (0.29-1.05) |
| Obesity | 1.09 (0.84-1.42) | 1.20 (0.83-1.73) |
| Per chronic diseases | 0.89 (0.75-1.06) | 1.15 (0.91-1.48) |
| **Unhealthy behaviors** |  |  |
| Current smoking | NA | NA |
| Problem drinking | NA | NA |
| No exercise | **2.05 (1.53-2.75)** | **1.50 (1.06-2.13)** |
| Per unhealthy behavior | 1.13 (0.93-1.38) | 1.02 (0.77-1.34.) |
| The KHPS had questionnaires of weekly working hours in 2009, 2011,2012, and 2013, long working hours defined as over 52 hours per week using last questionnaires of weekly working hours in each follow-up year.  This model excluded participants who had an event of CVD before long working hours during follow-up periods.  Bolds are statistical significance.  Adjusted model: adjusting for age, educational level, household income level, type of work, and occupational classification.  NA: Not available due to insufficient number of cases in the strata to construct the Cox-proportional hazard model.  All results were from referred counter group of each categories (no hypertension, no diabetes, no dyslipidemia, no obesity, past/never smoking, social/never drinking, or regular exercise). | | |
